# Droplet Motion Driven by Liquid Dielectrophoresis in the Low-Frequency Range

**DOI:** 10.3390/mi15010151

**Published:** 2024-01-19

**Authors:** Sarah Günther-Müller, Raschid Azizy, Steffen Strehle

**Affiliations:** Institute of Micro- and Nanotechnologies (IMN) MacroNano®, Microsystems Technology Group, Technische Universität Ilmenau, Max-Planck-Ring 12, 98693 Ilmenau, Germany; raschid.azizy@tu-ilmenau.de (R.A.); steffen.strehle@tu-ilmenau.de (S.S.)

**Keywords:** microfluidics, fluid manipulation, droplets, liquid dielectrophoresis, LDEP

## Abstract

Electrohydrodynamic wetting manipulation plays a major role in modern microfluidic technologies such as lab-on-a-chip applications and digital microfluidics. Liquid dielectrophoresis (LDEP) is a common driving mechanism, which induces hydrodynamic motion in liquids by the application of nonhomogeneous electrical fields. Among strategies to analyze droplet movement, systematic research on the influence of different frequencies under AC voltage is missing. In this paper, we therefore present a first study covering the motion characteristics of LDEP-driven droplets of the dielectric liquids ethylene glycol and glycerol carbonate in the driving voltage frequency range from 50 Hz to 1600 Hz. A correlation between the switching speed of LDEP-actuated droplets in a planar electrode configuration and the frequency of the applied voltage is shown. Hereby, motion times of different-sized droplets could be reduced by up to a factor of 5.3. A possible excitation of the droplets within their range of eigenfrequencies is investigated using numerical calculations. The featured fluidic device is designed using larger-sized electrodes rather than typical finger or strip electrodes, which are commonly employed in LDEP devices. The influence of the electrode shape is considered simulatively by studying the electric field gradients.

## 1. Introduction

Electrohydrodynamic (EHD) control enables the modification of the wetting properties of liquids on solid surfaces through the application of electrical fields. Numerous applications benefit from the resulting control of droplets on planar surfaces, such as display technology [1,2,3,4], optics [5,6,7,8] and lab-on-a-chip applications [9,10,11], just to name a few.

Liquid dielectrophoresis (LDEP) represents a fundamental EHD driving principle that allows the movement and manipulation of liquids in the range of very small volumes from micro- to nanoliters [12,13]. LDEP induces a bulk liquid motion through the application of a nonhomogeneous electrical field to liquids with dipoles. LDEP is phenomenologically similar to particulate dielectrophoresis as polarizable liquids are drawn into regions of higher field strength but with the further effect that their shape is also influenced by the electric field [12]. The benefits of LDEP-driven microfluidic control, including reversibility, low operation voltages and the fact that no moving parts are required, make it an intriguing EHD driving principle [14,15,16,17]. LDEP forces can be applied not only to perfectly dielectric liquids but also to many liquids that have the properties of both a conductor and a dielectric [18]. Over the past decade, a novel LDEP configuration was established where the bulk force is concentrated on the three-phase contact line through interdigitated coplanar electrodes [18,19,20,21,22]. This concentration leads to a change in the contact angle of the liquid rather than a bulk liquid motion [19], which can be expressed by

(1) $$\cos\theta_{\mathrm{DEW}}\left(V\right)=\cos\theta_{0}+\frac{\epsilon_{0}\left(\epsilon_{L}-\epsilon_{A}\right)}{2\delta \gamma_{\mathrm{lv}}}V^{2}$$

where $\epsilon_{L}$ and $\epsilon_{A}$ are the relative permittivities of droplet liquid and ambient fluid, respectively, and δ is the penetration depth of the electric field into the liquid. The term “dielectrowetting” (DEW) was introduced to distinguish this setup from classic LDEP actuation. DEW gained attention for its potential to overcome the limitations of electrowetting (EW), another EHD driving mechanism. In EW, the apparent angle of a conducting droplet can be modulated by the application of the potential between the droplet and the conducting surface, which are separated by a thin insulating layer in the electrowetting-on-dielectrics (EWOD) configuration. EWOD can only be applied on conducting liquids, whereas DEW can be employed on both conducting and dielectric liquids, depending on the frequency of the driving voltage. Furthermore, it was demonstrated that contact angle saturation, as known from EWOD actuation, is not present during wetting manipulation by DEW [22].

EWOD is usually conducted at DC or low-frequency AC voltages, whereas for LDEP, higher-frequency voltages are employed. Recent studies focus on LDEP-driven motion at driving frequencies from 1 kHz to 100 kHz [23], respectively, and EWOD-driven motion at frequencies from 10 to 50 Hz [24]. To date, no systematic study of the behavior of LDEP-driven droplets in the low-frequency range (below 1 kHz) is known. However, this range is of particular interest for biological samples as they can be damaged by Joule heating due to interaction with high-frequency fields [18,25].

In this paper, we address the low-frequency range and demonstrate LDEP-driven droplet motion for liquids with different permittivities. The experimental results show that droplet motion can be achieved at voltages down to root mean square (RMS) voltages $V_{\mathrm{RMS}}=16.25\;V$ . Furthermore, we show how actuation can be further improved by suitably adjusting the electrode contour to maximize the asymmetry of the electric field. By this measure, the total number of electrodes required can be reduced by using alternative layouts rather than the typical finger and strip electrodes.

## 2. Microfluidic System for LDEP Actuation

### 2.1. System Configuration

**Figure 1:**
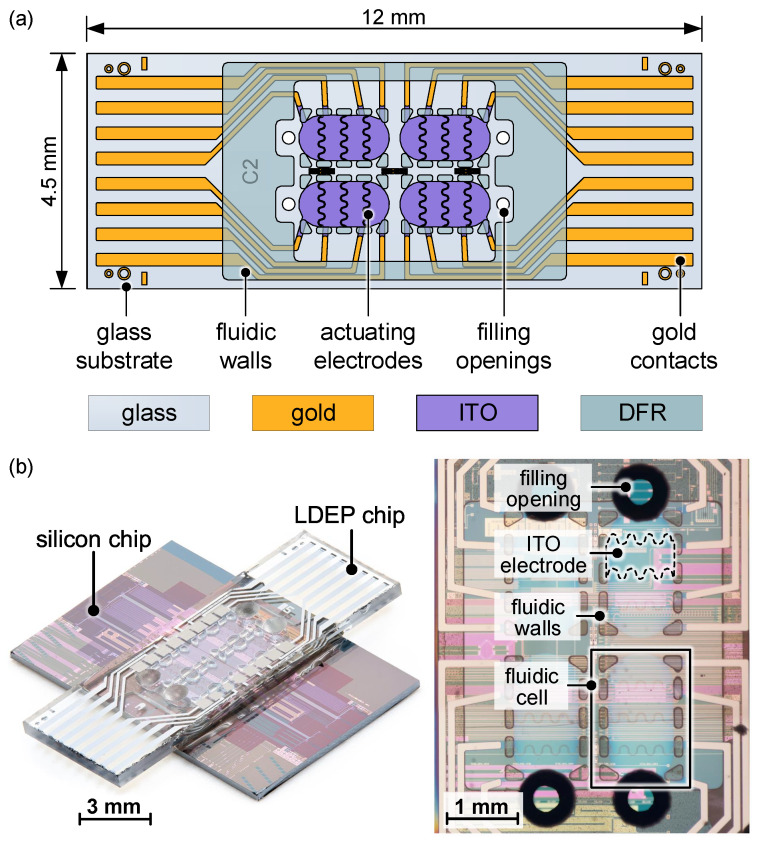
Configuration of LDEP system for analysis of droplet motion: (**a**) Layout of the fluidic cell based on glass substrate with ITO electrodes. Fluidic walls are defined by Ordyl SY300 dry film resist (DFR), and the cell is covered by an insulating and dewetting polymer coating. (**b**) Photograph of assembled device (left) and microscope image of the fluidic cell with relevant components (right).

The EHD system is built in a planar configuration comprising all fluidic components on one substrate. Thus, it can be directly used without an additional top cover or with a second substrate serving as sealing. This enables full flexibility of use and straightforward access to the droplet in contrast to configurations where the liquid is sandwiched between electrodes. The planar system also offers further benefits in terms of fabrication, liquid system exchange and maintenance through direct access. Regarding efficiency, parallel-plate configurations hold the advantage, as the electric field penetrates the entire droplet rather than just fringe fields on the surface. This can, however, be improved by efficient electrode designs, as discussed in Section 2.3. The dimensions are specifically aligned with an application in the field of optical switching devices, but this is not of further interest in this study [26]. Droplet and electrode sizing are in the typical range employed in microfluidic systems and are therefore suitable for further analysis of the droplet behavior. The device holds four fluidic cells. Each cell is 1730 μm × 1160 μm in size and consists of a chamber providing two positions for the droplet, see Figure 1a. Droplets having a diameter in the range of 300–900 μm can be inserted, which corresponds to volumes of around 7–70 nL . Each cell is equipped with four indium tin oxide (ITO) electrodes arranged in a coplanar configuration that are separated by a 20 μm gap in a wave-shaped form (see Section 2.3). The droplet motion is guided by so-called fluidic barriers with integrated backflow channels supporting the dynamic switching mechanism. The fluid walls are made of dry film resist (DFR, Ordyl SY300) with a thickness of 100 μm as previously utilized elsewhere in a comparable manner for microfluidic applications [27,28]. The fluidic device and the complementary substrate, which may contain, e.g., components for analysis, are assembled in a crossed configuration to ensure that all connections are conveniently accessible (Figure 1b).

To enable droplet movement, the surfaces of both substrates are required to be covered by a dewetting layer. Without such a coating, the droplet will stick to the surface, and the LDEP-driven motion will be hindered. The LDEP substrate is therefore covered by a polymer stack consisting of a dielectric to prevent current flow between electrodes and liquid and polytetrafluoroethylene (PTFE). The nonfluidic substrate is covered by PTFE as well. If for some reason ultrathin coatings are required, perfluorodecyltrichlorosilane (FDTS), a well-known antisticking material which is used, for instance, in nanoimprint lithography, can be alternatively applied as the dewetting layer [29].

### 2.2. Liquids

A biphasic liquid system comprising dielectric liquids with a high difference in their relative permittivities is introduced as this is essential for the actuation by LDEP, see Equation (1). The liquids ethylene glycol (EG) or glycerol carbonate (GC) are employed as droplet liquid and diphenyl sulfide (DS) as ambient liquid.

The ambient liquid DS has a relatively low permittivity of 5.4 and the droplet liquids EG and GC a high permittivity of 37.7 and 109.7, respectively. The properties of the liquids are listed in Table 1. Another important parameter is the dynamic viscosity of the fluids, as this determines the viscous stress of the fluid flow during movement. To allow an adequate droplet motion, a low viscosity of the ambient and droplet liquids is beneficial. In addition, the liquids have a high difference in their refractive indices, which makes them also suitable for use in optical switching systems [30].

### 2.3. Electrode Geometry

Unlike common strip [14,31] or interdigitated [18,19,32] electrode designs, larger electrodes are employed in this particular design to enable LDEP-driven droplet transport. Thus, the total number of electrodes and complexity of the design can be reduced. As the LDEP force is caused by electrical polarization effects in inhomogeneous electric fields, it appears beneficial to modify the electrode contour with respect to larger electric field inhomogeneities. Consequently, electrodes with straight and wave-shaped contours were investigated and compared by means of numerical modeling regarding the electric field intensity and distribution. Due to its geometry, the wave-shaped structure was expected to generate a significantly more inhomogeneous field distribution.

**Figure 2:**
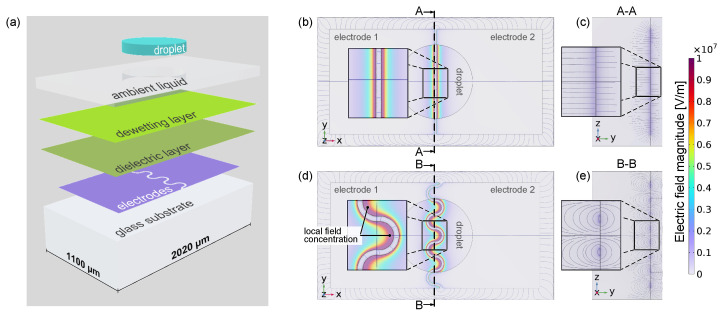
Numerical simulation model setup and obtained results concerning the investigation of the electric field distribution depending on the electrode gap morphology (straight vs. wave-shaped electrode layouts): (**a**) schematic illustration of the different material layers as used in the finite element method simulation model; (**b**) FEM-simulated electric field distribution for straight gap electrode layout in top view and (**c**) as corresponding cross section; (**d**) electric field distribution for wave-shaped gap electrode layout in top view and (**e**) as corresponding cross section.

To compare the electric field distribution that is generated by the electrodes, a steady-state electrostatic simulation based on the finite element method (FEM) is set up using the FEM software COMSOL Multiphysics 6.0. The 3D model comprises the electrode layer and surrounding materials including the liquids for an adequate representation of the spatial electric fields (Figure 2a). Materials and geometrical dimensions are aligned with the physical counterpart (more details are given in Section 4.2).

As evidently supported by the FEM results as shown in Figure 2b–e, a spatial increase in the magnitude of the electric field at the convex structures for the wave-shaped electrodes becomes apparent compared to straight electrode contours (see magnified insets in Figure 2b,d). Hence, a significant local increase in the field enhancing the LDEP driving force is obtained simply by changing the electrode geometry. The wave-shaped contour also provides significantly more inhomogeneous field areas compared to the straight gap contours, as can be seen from the *YZ* cross section of the electric field lines (Figure 2c,e). Hence, the wave-shaped electrodes were chosen for the fluidic device due to the provided advantages for LDEP actuation.

## 3. Results and Discussion

Droplet motion induced by LDEP actuation is analyzed using high-speed video imaging. To examine the influence of the driving voltage frequency on the droplet motion, the position, velocity and acceleration of the droplet during the transition between two positions are evaluated.

### 3.1. LDEP-Induced Droplet Actuation

**Figure 3:**
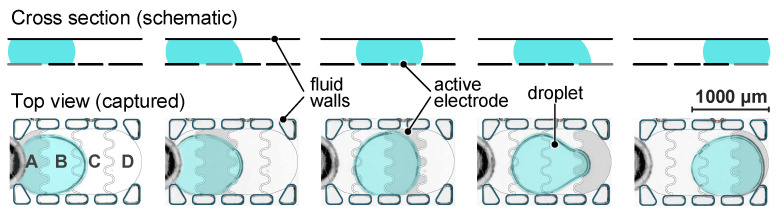
Droplet transport induced by sequential activation of coplanar LDEP electrodes: schematic sketch in cross-sectional and top view and captured frames of actual droplet movement (droplet highlighted in blue, electrodes and channel structures emphasized by image overlay).

To enable droplet transport, the coplanar electrodes are activated sequentially. The electrode arrangement leads to a concentration of the dielectrophoretic force resulting in an asymmetric change in the contact angle along the droplet contour, see cross section in Figure 3. This asymmetric deformation of the liquid meniscus leads to a pressure difference inside the droplet, initiating a bulk liquid movement [33].

To avoid charge trapping within the dielectric layers in the device [34], an AC voltage square wave is applied. Furthermore, the excited vibration of the droplet contour is assumed to help in overcoming the contact angle hysteresis [35]. The frequency of the driving voltage is adjustable in the range of 50 Hz –1600 Hz with peak-to-peak voltage $V_{\mathrm{PP}}=32.5\;V$ corresponding to a root mean square of $V_{\mathrm{RMS}}=16.25\;V$ . Since LDEP actuation is performed on nonconductive liquids, a low-frequency regime is utilized for actuation.

### 3.2. Motion Profile

To move the droplet in the cell from one position to another, adjacent electrodes (designated as A, B, C and D, see Figure 3) are actuated successively. To acquire the motion profile, a complete motion sequence back and forth between the two end positions is recorded. The sequence is captured with high-speed video imaging at 1000 fps. For the analysis, the displacement of one point of the droplet contour is tracked, and its position, velocity and acceleration are evaluated.

**Figure 4:**
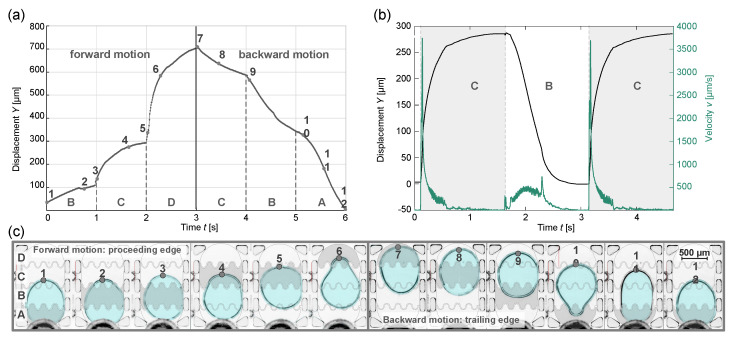
Analysis of droplet motion using high-speed video imaging: (**a**) Displacement *Y* of droplet contour over time *t*: complete motion sequence through sequential actuation of adjacent electrodes; (**b**) displacement and velocity graphs for droplet motion only between middle electrodes B and C where the droplet reaches its end positions asymptotically, which is evident from the velocity approaching zero and (**c**) captures of droplet motion through sequential actuation of adjacent electrodes in the fluidic cell. Active electrodes are emphasized by gray overlay, and designated positions correspond to the displacement graph in (**a**).

The motion curve with corresponding frames from the high-speed video clip is shown in Figure 4. The graph (Figure 4a) shows the displacement *Y* of the tracked point of the drop contour over time and an image sequence (Figure 4c) that corresponds to captures of the EHD cell at the designated points. Hereby, the deformation of the droplet during the different phases of the motion is recognizable. Active electrodes are indicated by a semitransparent gray overlay, and electrode and fluidic barrier outlines are emphasized by contour lines. The motion sequence is divided into two sections: the forward motion where the tracked edge is actively pulled by the LDEP force (proceeding edge) and the backward motion where the edge is passively dragged (proceeding edge). By considering the droplet outline, its position and its displacement, it becomes clear that the position changes considerably slower during the transition from one active electrode to the next. For example, this behavior can be seen between points 4 and 5 in Figure 4a. From this observation, it becomes apparent that the droplet approaches its final position asymptotically, which results in plateaus in the motion curve at each electrode transition. The monitored characteristics are valid for both directions of motion, whereby the trailing edge has a delayed response as expected due to viscosity and fluidic friction with the ambient liquid.

To evaluate the influence of the driving regime on the motion characteristics, the droplet is only excited to move between the two center electrodes B and C. Thus, a simple backward-and-forward movement is realized, and the motion stops when the droplet covers the active electrode. As there is no transition between electrodes for the respective motion directions, there are no flattenings in the curve that could distort the analysis, as shown in the corresponding distance and velocity plots (Figure 4c). Regarding the motion curves for different actuation regimes (Figure 4b,c), comparable curve characteristics become apparent. The velocity reduces gradually as the droplet approaches its end position. As subsequent electrodes are activated, droplet motion is induced, and the velocity increases steeply. After the peak value is reached, the speed drops continuously and becomes zero.

These observations are consistent with the droplet behavior reported by Cooney et al. in a comparable setup with coplanar electrodes [36]. The configuration with no direct electrical contact to the droplet is referred to as “ungrounded” EWOD. It is reported that nongrounded droplets will move halfway between activated electrodes and that the translational velocity goes towards zero as it approaches its final position. Following the definitions of EWOD and LDEP as discussed in Section 1, the described actuation setup leads to manipulation by means of LDEP since EWOD actuation requires direct electrical contact to the liquid [20]. Regardless of this matter of designation, the observations on droplet velocity from Cooney et al. [36] are consistent with the findings made here about LDEP actuation.

### 3.3. Frequency Variation

To investigate the dependence of the droplet motion on the frequency of the driving square wave voltage, actuation is again only carried out between the center electrodes B and C at constant voltage $V_{\mathrm{RMS}}\;=\;16.25\;V$ (see Figure 4c for motion curve). The electrode-on time is set long enough so that sufficient saturation time is provided for the droplet to obtain the maximal displacement.

**Figure 5:**
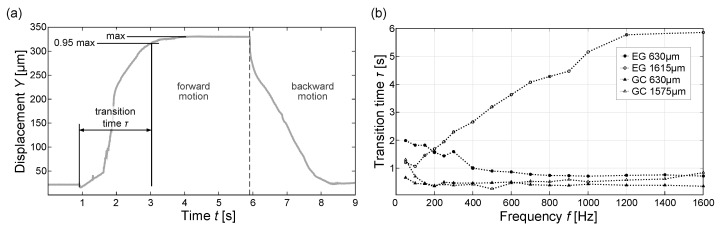
Investigation of the frequency dependence of the droplet motion for ethylene glycol (EG) and glycerol carbonate (GC) with diphenyl sulfide (DS) as ambient liquid: (**a**) definition of transition time τ as time span between start of the motion and reaching 95% of the maximum displacement in the forward motion where the droplet edge proceeds, and (**b**) transition time depending on excitation frequency for different-sized droplets of EG and GC (single measurements with connection lines).

The asymptotic approach of the droplet towards its end position makes it rather difficult to precisely determine the final position. To obtain the time span for the droplet motion, the average final value of the *Y* coordinate of the motion is determined (Figure 5a) and used to calculate a characteristic time. The so-called transition time τ is here defined as the time span between the start of the motion, which can be determined precisely, and the attainment of 95% of the final position value. This time constant provides a common basis for the evaluation of the droplet motion at varying excitation frequencies.

Based on this approach, the motion of different-sized droplets of EG and GC was analyzed in the frequency range from 50 Hz to 1600 Hz . For each liquid, droplets with a diameter of 630 μm and a length of 1575–1615 μm were analyzed. For each data point, a series of five to six motion sequences was recorded and averaged. The thus obtained graphs of the time constant versus excitation frequency are shown in Figure 5b. From this first study, it is clearly visible that the frequency has an impact on the achieved transition time. This influence seems to vary for the different droplet liquids. Furthermore, the droplet size apparently also affects the response. As shown in Figure 5b, the transition time for a 630 μm droplet of EG decreases with increasing frequency. For a droplet with a length of 1615 μm , however, the behavior can be observed to be exactly the opposite. In both cases, τ saturates at a final value. In contrast, for GC, both the larger and the smaller droplet show similar behavior. With increasing excitation frequency, the transition time decreases and seems to saturate at a minimum value. Furthermore, the difference between $\tau_{\min}$ and $\tau_{\max}$ is noticeably smaller compared to EG. The respective maximum and minimum transition times for the studied liquids and droplet sizes are summarized in Table 2. Here, the strong dependence of the large EC drop is clearly indicated by the ratio between $\tau_{\min}$ and $\tau_{\max}$ , which is larger by a factor of two than in the other cases. Overall, motion times for GC are lower than for EG, which shows a stronger response to a frequency variation. Furthermore, larger droplets show a stronger frequency dependence compared to smaller droplets. The impact of the droplet size on the transition time gives an indication that there might be a correlation between the excited vibration and the eigenfrequency of the droplet.

### 3.4. Numerical Model of Droplet Contour Vibration

To investigate the correlation between droplet size and transition time, a numerical model was established to study the vibration of the droplet contour at different excitation frequencies. Due to the nonlinear behavior of the system caused by fluid–fluid interaction described by the Navier–Stokes equation and the geometrically fixed position of the droplet in a constraint position, an analytical calculation of the eigenfrequency is not applicable here.

For the assessment of the oscillatory response of the droplet at various excitation frequencies, a 2D model was built, again using the FEM software COMSOL Multiphysics 6.0 and laminar flow physics. In the simulation, a cross section of the droplet with the length being equal to the diameter of the experimental setup was modeled. The droplet was squeezed between two parallel plates representing the LDEP substrate and cover plate, respectively. Further details about the the model design, the parameters and the boundary conditions are given in Section 4.6.

**Figure 6:**
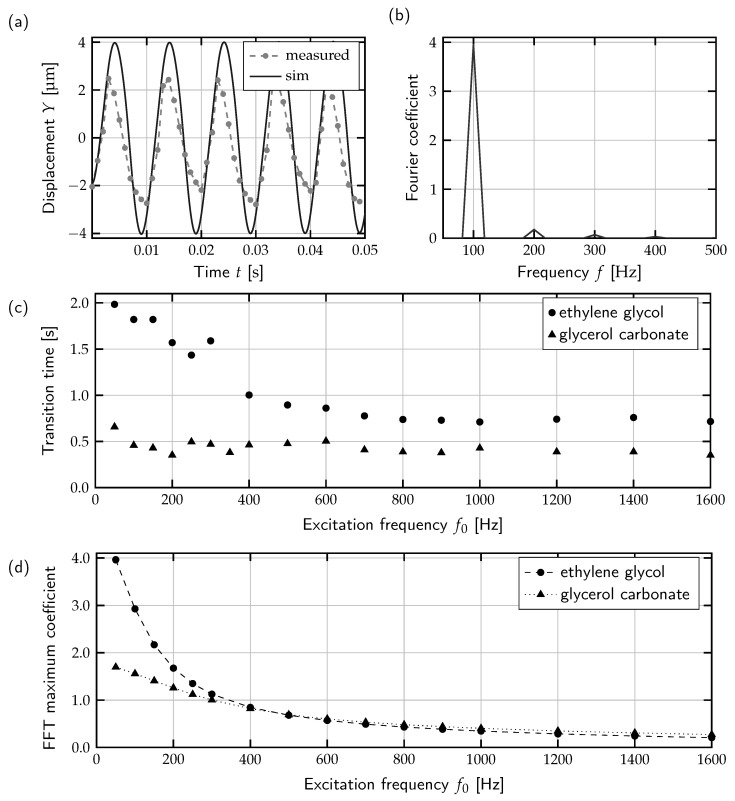
Numerical calculation of droplet contour vibration: (**a**) measured and simulated displacement at an excitation frequency $f_{0}=$ 50 Hz and (**b**) corresponding FFT response, (**c**) measured transition time depending on excitation frequency for droplets of EG and GC with a diameter of 630 μm and (**d**) peak values of FFT response for droplet contour vibration of EG and GC with a diameter of 630 μm .

For validation, simulated and measured droplet contour oscillation were compared. To acquire a measurement curve comparable to the simulated droplet vibration, the droplet was centered over an activated electrode so that the actuation resulted in an oscillation along the droplet contour. This vibration was captured with high-speed video imaging at an image rate of 1000 fps. In this way, the oscillation curve for an excitation frequency of 50 Hz was acquired. The measured and simulated droplet displacements over time are shown in Figure 6a. Based on the progression of the graphs, it can be seen that the measured and simulated vibration frequencies are in sufficient agreement. Thereby, the determined oscillation frequency corresponds accurately to the double of the excitation frequency. Since the contact angle under LDEP actuation depends on the square of the applied voltage (see Equation (1)), the excited vibration oscillates with $2\;f_{0}$ . Additionally, an asymmetry in the oscillation is noticeable in the experimentally obtained curve. This is a consequence of the difference between the motion directions in which the droplet once actively pulled and once passively dragged. Considering the absolute values, a difference between simulated and measured amplitudes is noticeable. The decrease in the amplitude with increasing frequency in experimental and numerical results could be explained by the viscous damping caused by fluid–fluid interaction. The deviation in the form of higher numerical values is due to the fact that fluidic friction is not considered in the model. In summary, the model reproduces the experimentally determined droplet oscillation in a reasonable approximation. To extract the frequency response of the calculated droplet vibration, a discrete Fourier transformation (DFT) was applied to the signal. To avoid aliasing, the sampling rate and interval were set to *T*/10,000 and 6T , respectively, where *T* is the period of the excitation frequency $T=1/f_{0}$ . Transformation and data analysis were carried out using the MATLAB R2021a numeric computing platform. The DFT of signal *x* was computed using a fast Fourier transform (FFT) algorithm. The FFT function is based on a library called FFTW [37]. To prevent leakage, a Hanning window was applied to the signal.

The resulting FFT response for the calculated vibration at an excitation frequency of $f_{0}=$ 50 Hz is shown in Figure 6b. The highest response can be found at a frequency of 100 Hz and then with a significantly reduced amplitude at the integer multiples of this value. This result agrees with the obtained vibration curve (Figure 6a), which oscillates at the frequency with the highest FFT response. For determining the FFT responses over the entire measured frequency range, the FFT is calculated separately for each excitation frequency for the droplets with a diameter of 630 μm (see Figure 5b). As the simulated system shows nonlinear behavior, since the motion between the liquids is described by Navier–Stokes differential equations, it is not possible to obtain the step response over the entire frequency spectrum in a single step.

The respective peak values of the response curves from the frequency sweep are shown in Figure 6d for EG and GC. For both graphs, the course of a typical attenuation curve can be seen with increasing frequency. This course generally meets the expectation since the oscillating amplitude decreases at increasing frequencies due to the Navier–Stokes interaction between the liquids. Regarding the amplitudes of the FFT, it becomes clear that the response for EG is considerably stronger than for GC. This is consistent with the experimental observations indicating that EG is more sensitive to a frequency change regarding the transition time (see Table 2). Comparing the FFT responses (Figure 6d) with the experimentally determined transition time curves (Figure 6c), an analogy in the course becomes clear. However, the measurement of the droplet motion time is only comparable to a limited extent with the vibration of the contour of a stationary droplet. Despite the limitations of comparability, the simple numerical model presented here is a suitable approach for making qualitative predictions about the behavior of a droplet under the control of different excitation frequencies.

## 4. Methods

### 4.1. Background of the Used Device Design

The used EHD device was originally configured for application in a fiber-optic switch, whose design is presented elsewhere [30,38]. The microfluidic system of the switch comprises a unique biphasic liquid combination actuated with LDEP, and the optical device consists of a photonic integrated circuit based on adiabatic waveguide couplers [39,40,41]. By changing the refractive index of the cladding material of one of the branches, the coupler changes its state from cross to bar state. Here, the required change in the effective index is achieved through an exchange of the liquid covering one branch of the broadband coupler via EHD forces.

### 4.2. Electric Field Simulation

The setup for the electric field simulation includes a bottom glass of EAGLE XG, the electrodes with wave-shaped or straight contours, a dielectric layer of Exilis, a dewetting layer of PTFE, a compressed droplet of ethylene glycol and the compressed ambient medium (diphenyl sulfide), see Figure 2a. All generated geometric regions are assigned a thickness and relative permittivity according to Table 3 corresponding to the physical setup.

The boundary conditions for the geometric outer surfaces of the simulation model are zero charge, and the various electric potentials of the solid bodies are set to zero as initial values. The electrode is inserted in a two-dimensional manner to reduce calculation time, and the potential between the two electrodes is chosen to be zero volts for the left electrode and 35 volts for the right electrode. For meshing, the simple physics-driven tetrahedral mesh of element size “finer” is chosen. For the comparison of the two electrode shapes, the magnitude of the electric field is considered.

### 4.3. LDEP Device Fabrication

**Figure 7:**
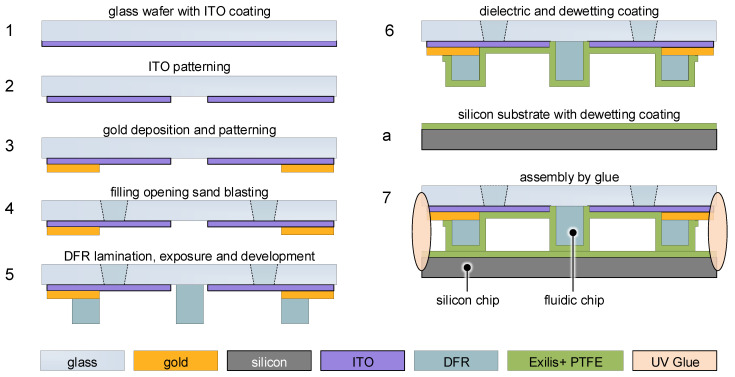
Schematic of fabrication flow of LDEP device for optical switching.

The fabrication of the EHD cells was carried out at the technology center of the Institute of Micro- and Nanotechnologies (IMN). In Figure 7, the fabrication flow is shown. Base substrates are Eagle XG^®^ glass wafers carrying 60 nm of ITO (1). In the first step, the ITO electrodes are patterned by a wet chemical etching process (2). Then, 400 nm gold for contact pads is deposited and subsequently structured, also by wet chemical etching (3). A thin layer of chromium (30 nm ) serves as adhesion promoter between gold and ITO/glass. In the next step, the filling openings are generated by a powder-blasting process (4). The fluid walls (100 μm ) are obtained by double lamination and photolithographic patterning of Ordyl SY355 permanent dry film resist (5). The entire fluid chamber is covered with Exilis dielectric (300 nm ) and PTFE (50 nm ) as dewetting layer, both deposited at room temperature from vapor phase by a commercial supplier (6). Areas not to be coated are covered with tape during deposition. The optical substrate is covered with FDTS as dewetting layer from vapor phase (a). In the concluding step, LDEP and optical substrate are assembled by UV-curing glue (7).

### 4.4. Device Assembly and Filling

**Figure 8:**
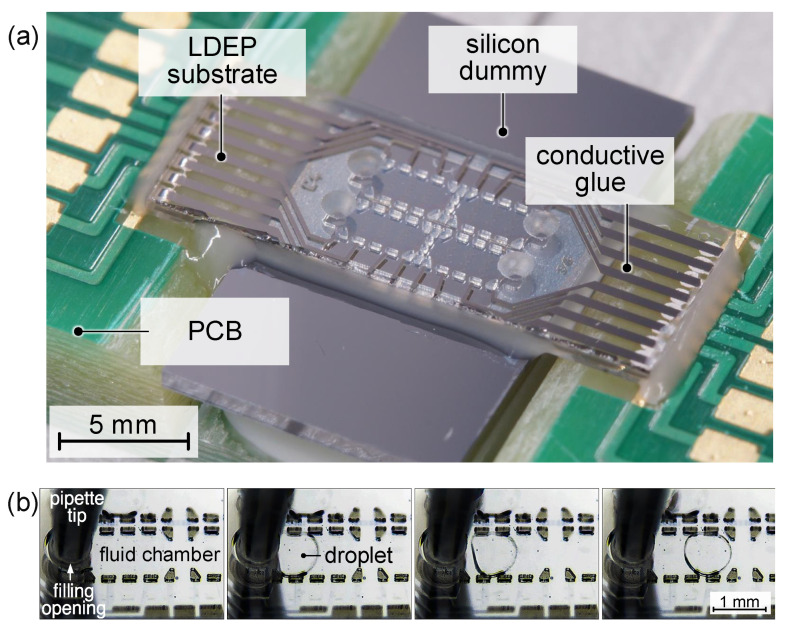
LDEP substrate assembly and filling: (**a**) experimental setup with LDEP device with assembled silicon dummy covered by dewetting coating on PCB and (**b**) captured frames of droplet filling sequence where a droplet of ethylene glycol (EG) is pushed into the fluidic chamber which was vacuum-filled with diphenyl sulfide (DS) by pipette tip connected to a pumping device.

LDEP and optical sample are aligned and assembled together using a micromounting tool. Bonding and fluidic sealing are obtained by application of the UV-curing glue DELO^®^ Katiobond^®^ LP655 along the outline of the fluidic cell. The adhesive features a superior chemical resistance and is thus stable to the applied liquids. For the experimental investigation of the behavior of the liquids under LDEP actuation, PTFE-coated silicon dummy chips are used instead of optical substrates due to their limited availability. As the surface is covered by a dewetting coating, this modification has no impact on the LDEP response of the liquids compared to a photonic integrated circuit with identical coating. Electrical contact with the printed circuit board (PCB) is made by conductive adhesive, see Figure 8a.

The liquids are filled manually after assembly of the cell. The filling of the ambient liquid is supported by vacuum. Hereby, a droplet of the liquid is placed on top of the chip so that all openings are covered. Subsequently, the substrate is placed in a vacuum chamber, which is evacuated to a pressure of 0.1 mbar for 30 s . After the chamber is flooded with air again, the ambient liquid is pushed into the fluidic channels. This procedure has proven to be the best technique for ambient liquid filling, as it effectively avoids air bubbles in the chamber, unlike other methods.

Dosing of the droplet liquid is performed via a high-precision syringe pump (Cetoni low Pressure Syringe Pump neMESYS 290N) connected to a pipette tip which is precisely moveable in three axes. The droplet liquid is injected into the opening and subsequently flushed into the fluidic cell, see Figure 8b. Using this method, droplets of different sizes in the range of 30 nL –100 nL can be generated.

### 4.5. Experimental Setup

The droplet motion is captured with high-speed video imaging. For that purpose, a high-speed microscope setup by Keyence (VW-9000 with zoom lens VH-Z20R) is employed. The recorded droplet motion is analyzed via digital image processing. Thereby, the commercial software motion analyzer VW-9000 by Keyence is used. The motion characteristic is evaluated with respect to droplet position, velocity and acceleration at different points along the droplet contour.

The control electronics to actuate the LDEP device were developed in cooperation with Bartels Mikrotechnik GmbH. The frequency of the rectangular voltage is adjustable in a range from 20 Hz to 1600 Hz with peak-to-peak voltage $U_{\mathrm{PP}}$ up to 50 V . The electronic system allows the parallel control of up to 64 outputs, whereby the 2 × 2 cell requires a maximum of 16 electrodes. All outputs can be controlled independently from each other with a special feature allowing the simultaneous control of two or more electrodes with a signal shifted by half a period ( π ).

### 4.6. Droplet Contour Vibration Model Setup

**Figure 9:**
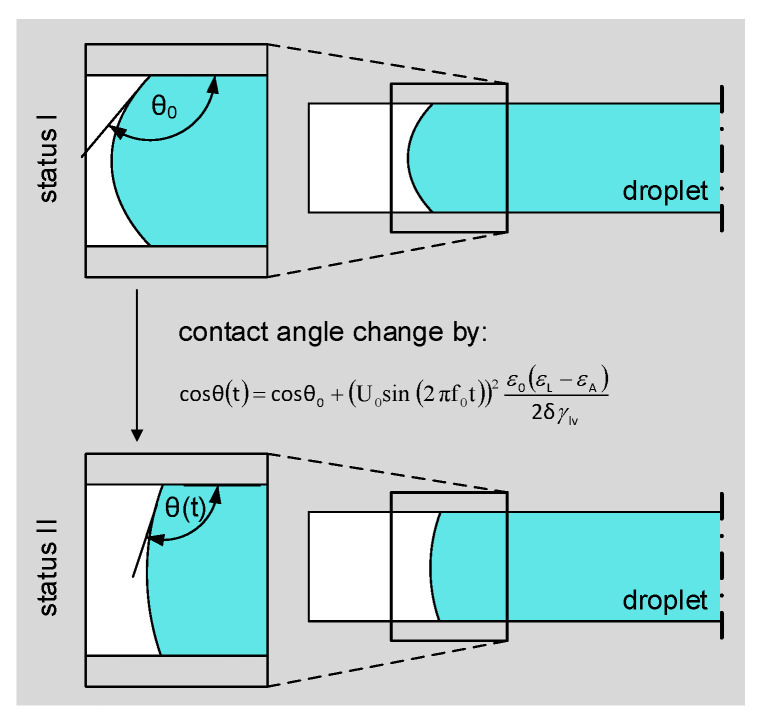
Schematic sketch of numerical simulation to model the droplet contour vibration excited by dielectrowetting actuation.

The numerical model to determine the droplet contour displacement is set up using COMSOL Multiphysics 6.0 as 2D cross section across the droplet, see Figure 9. Blue areas are assigned to droplet liquid properties, white areas to ambient liquid properties. All parameters used in the model are listed in Table 4.

Fluid–fluid interaction is modeled by the laminar flow physics interface. The contact angle is defined at the three-phase contact point between wall and droplet by the wall fluid interface node. This node defines the position of the fluid–fluid interface and enables tracking of its progression. The contact angle change driven by dielectrowetting is specified according to Equation (1) time-dependently via the applied voltage, as follows:

(2) $$\cos\theta_{DEW}\left(V\right)=\cos\theta_{Y}+{\left(V_{0}\;sin\left(2\pi f_{0}t\right)\right)}^{2}\;\frac{\epsilon_{0}\left(\epsilon_{L}-\epsilon_{A}\right)}{2\delta \gamma_{\mathrm{lv}}}$$

Top and bottom wall boundary conditions are set to no slip and the left and right walls as outlets to allow ambient liquid flow. At the dot-dashed line in Figure 9, the boundary condition symmetry was applied. For each excitation frequency *f*, a time-dependent study with a total time of $T_{\mathrm{tot}}=6T$ at time steps $t_{\mathrm{st}}=T\times 10^{-4}$ with T=1/f0 was carried out.

## 5. Conclusions

We reported an investigation of the response of dielectric liquids under LDEP activation in a low-frequency regime of the excitation voltage. A substrate with coplanar electrodes was used for this purpose. Due to the planar electrode configuration, the LDEP force was focused near the surface of the substrate and thus to a three-phase contact line of the droplet whereby a decrease in the contact angle was observed under actuation as it is known from the electrowetting effect. Droplet transport was induced by sequential actuation of adjacent electrodes with optimized contours supported by numerical simulations. Alternative reported devices to implement droplet transport by means of LDEP used strip electrodes [14,31], parallel-plate electrode configurations [42,43] or coplanar electrodes divided into subelectrodes [44]. The employed fluidic system thus represents the first device enabling droplet transport by LDEP forces over a planar surface with simple adjacent electrodes to the best of our knowledge.

Driving voltages of the rectangular signal for the droplet motion were $V_{\mathrm{RMS}}=16.25\;V$ , corresponding to a peak-to-peak voltage of $V_{\mathrm{PP}}=32.5\;V$ and thus considerably lower than reported for comparable LDEP devices [14,31,43,44].

Analysis of the droplet motion profile showed that the velocity converges to zero as the droplet reaches its end position. Thus, the droplet approaches its final position asymptotically. The focus of the experimental investigation is set on the frequency dependence. For this purpose, the motion of equal-sized droplets of EG and GC was analyzed and compared over a frequency range of 50 Hz –1600 Hz . The transition times of EG droplets could be reduced by a factor of 2.8–5.3 depending on the droplet size and for GC by a factor of 1.9–2.5. Hence, EG reacts more strongly to a change in frequency than GC and, for both liquids, larger droplets react more strongly than smaller ones.

To examine the eigenfrequency of the droplets as a possible cause of the varying transition times over the frequency range, the resulting oscillation of the droplet contour under symmetrical excitation was simulated numerically, and an FFT of the resulting displacement was performed. It became apparent that EG reacts more strongly to a change in the excitation frequency according to the Fourier coefficient, which corresponds to the measured results. This numerical simulation provides a simple tool that allows qualitative predictions about the behavior of droplets of different sizes and liquid materials under the control of different excitation frequencies.

Overall, it can be concluded that a variation in a low-frequency range of 50 Hz to 1600 Hz already has a significant effect on droplet motion. To the authors’ knowledge, so far, no comparable study on the behavior of liquids under EHD actuation in the low-frequency regime has been carried out. Instead, focus has been mainly set on the high- and low-frequency limits corresponding to the difference between LDEP and EWOD actuation [45,46,47,48,49]. The obtained data encourage further investigation with different-sized droplets and in an extended frequency range of up to ∼5 kHz .

## Figures and Tables

**Table 1:** Properties of system liquids.

Property	Ethylene Glycol (EG)	Glycerol Carbonate (GC)	Diphenyl Sulfide (DS)
CAS no.	107-21-1	931-40-8	139-66-2
Linear formula	C_12_H_10_S	C_2_H_6_O_2_	C_4_H_6_O_4_
Relative permittivity	5.4	37.7	109.7
Surface tension ( $\mathrm{mN}m^{-1}$ )	41.8	48.2	57
Density (g/cm^3^)	1.11	1.19–1.23	1.11
Dynamic viscosity ( mPas ) (25°)	4.2	17.4	85.4

**Table 2:** Transition times (minimum and maximum values) as derived from frequency variation measurements depending on the used liquid and droplet size.

Droplet	Size ( μ m)	$\tau_{\max}$ (s)	$\tau_{\min}$ (s)	$\tau_{\max}/\tau_{\min}$
Ethylene	1615	5.9	1.1	5.3
glycol	630	2.0	0.7	2.8
Glycerol	1575	1.3	0.5	2.5
carbonate	630	0.7	0.4	1.9

**Table 3:** Electrical field simulation: relative permittivities of materials.

Geometric Area	Material	Relative Permittivity	Thickness
Bottom glass	Eagle XG^®^	5.27	500 μm
Dielectric layer	Exilis	2.6	300 nm
Dewetting layer	PTFE	2.1	50 nm
Droplet	Ethylene glycol	37.7	100 μm
Ambient liquid	Diphenyl sulfide	5.4	100 μm

**Table 4:** Parameters of the Comsol simulation model.

Name	Value	Description	Comment
$f_{0}$	50–1600 Hz	Excitation frequency	
$U_{0}$	16.25 V	Applied voltage, RMS	
*H*	102 μm	Height of the fluid channel	Determined with laser scanning microscope Olympus LEXT 4100
$L_{\mathrm{droplet}}$	630 μm	Droplet diameter	
$\gamma_{\mathrm{IFT},\mathrm{GC}}$	8.5 $\mathrm{mN}m^{-1}$	Interfacial tension between GC and DS	Determined with KrüssDrop Shape Analysis DSA10 contact angle measuring device
$\gamma_{\mathrm{IFT},\mathrm{EG}}$	0.9 $\mathrm{mN}m^{-1}$	Interfacial tension between EG and DS	
$\theta_{Y}$	130°	Initial contact angle	
$d_{\mathrm{Ex}}$	300 nm	Thickness of the Exilis dielectric layer	
$\epsilon_{\mathrm{Ex}}$	2.6	Relative permittivity of the Exilis dielectric layer	Value provided by manufacturer (GVD)
$d_{\mathrm{PTFE}}$	50 nm	Thickness of the PTFE layer	
$\epsilon_{\mathrm{PTFE}}$	2.1	Relative permittivity of the PTFE dielectric layer	Value provided by manufacturer (GVD)
$C_{\mathrm{tot}}$	63.6 $\mu Fm^{-1}$	Total capacity of Exilis and PTFE per unit area	
$\epsilon_{\mathrm{GC}}$	109.7	Relative permittivity of glycerol carbonate	See Table 1
$\epsilon_{\mathrm{EG}}$	37.7	Relative permittivity of ethylene glycol	See Table 1
$\epsilon_{\mathrm{DS}}$	5.4	Relative permittivity of diphenyl sulfide	See Table 1

## Data Availability

Dataset available on request from the authors.
